# Progress in Surgery

**Published:** 1900-08-25

**Authors:** 


### Progress in Surgery.

### CEREBRAL SURGERY.

**Cases of Brain Injury.**—Edmund E. Dyer[1] relates three interesting cases of this class. The first was that of a man of 20, who had been struck with the revolving handle of a steam winch. The blow had produced a wound 2¾ inches long, and extreme shattering of bone, with depression, at a distance of three inches above the left ear. The man had stertorous breathing and muscular twitching, and was insensible except to strong stimuli. There was a history of violent convulsions immediately after the accident. Trephining was performed and several pieces of bone removed. The dura mater was apparently uninjured. The frontal bone was elevated with the finger and went back into its place with a snap. Immediately after this the breathing became natural. The wound healed well. On the ninth day puffiness and redness developed over the nose and forehead, but subsided again in a few days, when he began to speak and answer questions, but cerebration was very slow. On the twenty-first a similar swelling developed in the occipital region. Then photophobia came on, and the patient lay curled up on his left side. He died comatose on the twenty-eighth day. At the post-mortem the brain was found to be more or less covered with pus, and immediately below the seat of injury was an abscess. This communicated with the lateral ventricle. In the right hemisphere was another abscess below the surface of the angular convolution. There was purulent basic meningitis. The absence of paralysis, the extraordinary destruction of brain tissue, and the extent of time the patient lived are remarked upon. Case 2 was an old soldier, aged 56, admitted to hospital for right hemiplegia. Cerebration was very slow. No definite history was obtainable. Eight days later he suddenly developed most violent convulsions, which subsided under heroic doses of chloral and morphia. He died on the twenty-fourth day. On making horizontal sections of the brain no less than seven clots were found in different parts of the brain, one being in the cerebellum, each of a different age. Three were in the left hemisphere, one high up in the fissure of Rolands, another in the middle frontal convolution, and the third in the occipital lobe. In the right hemisphere the three clots found were almost in exactly corresponding positions. The oldest clot was that in the left frontal region, the most recent in the left occipital. Dyer thinks the cerebellar clot was the cause of the convulsions. There was no affection of breathing. A third case is given, in which recovery followed destruction of a considerable amount of brain tissue.

**Cortical Excision for Traumatic Epilepsy.**—Dr. Brewer[2] brought forward an example of this at a meeting of the Philadelphia Academy of Surgery. Eight years previously there had been traumatism followed by epilepsy, which gradually increased in severity, and was finally accompanied by progressive paralysis of the muscles of the right arm and leg. In January, 1899, the man was having frequent attacks of *grand mal*, and was unable to use his right arm, and walked with great difficulty. Exploratory osteo-plastic resection was made over the left motor area. An extensive adhesion was found between the dura and cortex of the brain, caused by an extremely vascular inflammatory exudate. The wound was closed, and soon healed, and the paresis of the arm and leg improved. Six weeks after it was concluded that removal of the exudate, unless accompanied by an extensive cortical excision, would probably have no effect upon the epilepsy, as there were present evidences of cortical degeneration. Energetic anti-syphilis treatment was tried for two months, but the epileptic seizures increased in frequency (six to eight a day). An osteoplastic flap was again raised. The exudate was thinner and less vascular. It was removed, together with the underlying cortex, to the depth of five-eighths of an inch, the piece removed being about the size of a dollar. The hæmorrhage was easily checked, and the dura and bone-flap replaced. Almost complete paralysis of the arm and leg followed the operation, and the fits increased to such an extent that he would have from 40 to 50 a day. There was no infection of the wound. About ten days after the operation the fits ceased suddenly and he was free from them for two months. Since then he had only had ten fits in eight months. The paralysis had remained the same.

Dr. Kammerrer[3] advocates the use of Gigli's saw for separating the bones in osteo-plastic resection, and also the small trephine- and dural separator recommended by Dr. Buchanan in the *Medical Record* about a year and a half ago. Having made the small trephine openings the dural separator is introduced, and after that it is easy to pass an eyed probe from one trephine opening to another, and, with the aid of this, the wire of Gigli's saw can be easily passed. An inch to an inch and a half was the interval left between two trephine openings. Dr. Clarke considers the trephine openings made were too small, especially for the skulls of adults, and used a three-eighths inch trephine. The method was certainly a time saving one, and if the bones were thin the operation could be done in from 20 to 25 minutes. Herbert Marson[4] records a case of operation for traumatic Jacksonian epilepsy from the Staffordshire Infirmary. The patient was a boy of 10, who suffered from partial paralysis of the left arm and leg and frequent epileptiform attacks. Eight years previously he had fallen from a third storey window, injuring the right side of the head. He was picked up in an unconscious state. From that time there had been considerable weakness of the left arm, and to a less degree of the left leg. He could not put his hand to his head, and could only walk with difficulty. For three months he had been subject to fits, sometimes having three or four a day. They began in the left leg and were limited to the left side. They produced unconsciousness and great exhaustion. A slight depression in the skull existed on the right side. This was removed with a trephine. About two drachms of clear fluid then escaped. The under surface of the bone was rough, and a cyst the size of an olive was found there, adherent to the cranium. This was removed. For some days there was a discharge from the wound of large quantities of a non-albuminous fluid. By the tenth day it had healed. He had several fits during the first month, but they were much less severe than before the operation; and during the next three months there were only three fits. He had regained considerable power in the arm and leg, and could now put his hand on the top of his head and could walk much better.

[1] Lancet, June, 1900. [2] Annals of Surgery, April, 1900. [3] Annals of Surgery, Mar. [4] Brit. Med. Jour., June.

### GENITO-URINARY SURGERY.

**Prostatic Hypertrophy.**—The treatment of enlarged prostate is in the present day in an uncertain state. D. Wallace has reviewed much of the recent literature on the subject.[1] As regards drainage, the perineal route is preferred by Dandridge,[2] who thinks it safer with patients who have damaged kidneys. Septic infection is an important factor in the mortality of cystotomy. Poncet quotes 117 cases, with an average mortality of 25 per cent.,[3] the mortality being three times as great in cases with septic urine as in cases in which it was aseptic. This corresponds with Barling's statistics of 169 cutting operations for stone in the London hospitals. In cases drained by the perineal route, then the most efficient means of drainage, the mortality was only one-third that by the suprapubic route. Suprapubic drainage has, however, been much improved by the introduction of suction drainage, on the principle of the Sprengel air-pump.

**Vasectomy,** as a treatment for enlarged prostate, is to a considerable extent uncertain. In some cases the results are highly gratifying; in others, such as in carcinomatous or hard fibroid prostates, the results are disappointing. In yet another group there is some change in the physical condition of the bladder which minimises the effects of vasectomy. Reginald Harrison has considered these physical conditions from observations of prostates subsequent to a previous vasectomy.[4] In all the cases there was evidence that the prostate had shrunk as a result of the operation, although the natural powers of micturition were not regained. Harrison concludes that vasectomy induces shrinkage, which will facilitate the introduction of a catheter, but not necessarily restore expulsive power, particularly when it is dependent upon a sacculated and trabeculated bladder. Sacculation of the bladder is sufficient to throw the natural expulsive powers out of gear, and cases are quoted in support of this argument. The inference drawn from these observations is that if vasectomy were resorted to at an earlier stage, *i.e.*, at the stage when catheter life is usually commenced, then the expulsive power of the bladder might be retained. Moreover, Harrison reaffirms that the sexual power persists when vasectomy alone is performed, but is lost when the testicles are removed.

Bottini's operation has been revived and strongly advocated in America. Dr. Willy Meyer says the operation has "come to stay."[5] It may therefore be well to consider the recent facts of the case, particularly as the operation can be employed when vasectomy is useless. The cases, of course, require selection, as all cases of enlarged prostate do which require operative treatment. Prior to operation large doses of quinine are administered. The operation is performed as aseptically as possible, and the knife is carefully sterilised before use. The requisite electric current is obtained from the main with the help of an alternator and rheostat, or else from a storage battery specially charged and fitted with an amperemeter. To use Bottini's words, the operation requires adroitness and care in order to avoid annoying surprises. Rigid antisepsis must be the rule in operation. A soft, boiled catheter is partially introduced, the anterior urethra irrigated out, and then the catheter passed into the bladder. The bladder is then well irrigated, and sterilised eucaine solution is injected. The current should be sufficiently strong to make the knife white hot. The instruments are finally prepared, and after five minutes the eucaine solution is withdrawn from the bladder. Then the anterior urethra is treated with eucaine solution, and the instrument passed. The electric and iced-water connection are fixed, and "the prostate well hooked with the beak of the incisor exactly in the median line"—an important point. The right forefinger is pressed into the rectum to test the position of the beak of the instrument. The current is turned on of the desired strength, while the operator waits fifteen seconds for the knife to become properly heated. Three cuts are then made, one in the middle line and one in each lateral lobe, the median, and most important one, being made first. Auscultation of the suprapubic region is done meanwhile, in order to make sure of the working of the instrument by the "sizzling" sound. The cauterisation process takes ten minutes, and a final irrigation of the bladder is unnecessary.

If the patient begins to urinate voluntarily afterwards the treatment is simple, and the surgeon should not interfere. If, however, there be much swelling of the gland afterwards, with retention, or if urethral fever supervene, then difficulties arise. One should always avoid an anterior incision for fear of causing inflammation of the cave of Retzius and death, as occurred in one case. The patient must be watched for three weeks, since hæmorrhage is likely to occur during the second or third weeks. The cystoscope may first be used in all cases of doubt to determine the general shape of the projecting prostate. During the operation the bladder should be injected with an aseptic lotion, for if air be used instead there is risk of air-embolism of the inferior vena cava.

One of the most difficult points to determine in Bottini's operation is the exact length of the incision desirable, for the surgeon works in the dark. An estimate of the length of the prostatic urethra may be gained by measuring the excessive distance a catheter must pass before the flow of urine commences, also by rectal palpation and measurement with the forefinger. When, however, the glandular elements predominate or a pedunculated middle lobe exists then no certain estimate is possible. Meyer says: "The occurrence here mentioned may often put the operator into a most annoying position." In reading Meyer's paper one is struck by the many difficulties and dangers which present themselves, suggesting that the instrument may be a source of danger even in the best hands. In view of these dangers and the very great pathological variety of enlarged prostates, there are probably few surgeons who would willingly do Bottini's operation. The dangers are mainly as follows: (*a*) Perforation of the bladder, a part of the base of the viscus having been caught up by the beak of the instrument; (*b*) perforation of the urethra from too long an incision with the electric knife or an unexpectedly compressible prostate; (*c*) bending of the knife; (*d*) giving out of the battery; (*e*) urethral fever; (*f*) septicæmia and pyæmia; (*g*) pulmonary embolism; (*h*) absolute retention; (*k*) hæmorrhage; (*l*) painful spasms at the neck of the bladder — a common complication. Hæmorrhage, when it occurs, comes on in the second or third week, when the sloughs separate. Hence the surgeon should abstain from washing the neck of the bladder for the first few weeks, so as to lessen the danger of this. If painful spasms and inability to urinate be apparent some time after operation, they are indications of incomplete performance and of the necessity to repeat the operation. Recurrence is stated to be very rare.

With regard to the advantages of the operation, relief from the painful spasms and increase of the patient's weight are the chief. The chronic constipation is usually relieved, the symptoms caused by cystitis are removed, and in a proportion of the cases the turbidity of the urine disappears.

With regard to the indications for Bottini's operation opinions differ. Meyer thinks the operation should be done at the time when the patient usually commences catheter life, and that of all direct operative measures upon the prostate Bottini's operation is one which has the lowest mortality and the highest percentage of cures.

As to prognosis, Meyer has operated 30 times on 24 cases. In nine cases there was a lasting cure, and seven cases were much improved. There were two immediate and several remote deaths from the operation. Cures were about 30 per cent., improved cases rather under 30 per cent., and the mortality 16 per cent.

[1] Scottish Med. and Surg. Jour., Jan. 1, 1900, 77. [2] Ann Surg., July, 1899. [3] Acad. de Med., 1898. [4] Lancet, 1900, I. 1275. [5] Med. Rec., 1900, lvii., 705 and 793.

